# Outbreak of acute undifferentiated febrile illness in Kathmandu, Nepal: clinical and epidemiological investigation

**DOI:** 10.1186/s12879-020-4803-8

**Published:** 2020-01-30

**Authors:** Sunil Pokharel, Manan Karki, Bhim Acharya, Baburam Marasini, Amit Arjyal

**Affiliations:** 1 Patan Academy of Health Sciences, Lalitpur, Nepal; 2 Epidemiology and Disease Control Division, Ministry of Health and Population, Kathmandu, Nepal

**Keywords:** Outbreak, Undifferentiated fever, Clinical features, Epidemiological investigation, Risk factors

## Abstract

**Background:**

Outbreaks of acute undifferentiated febrile illness (AUFI) are common in Nepal, but the exact etiology or risk factors for them often go unrecognized. Diseases like influenza, enteric fever and rickettsial fevers account for majority of such outbreaks. Optimal diagnostic tests to inform treatment decisions are not available at the point-of-care. A proper epidemiological and clinical characterization of such outbreaks is important for appropriate treatment and control efforts.

**Methods:**

An investigation was initiated as a response to increased presentation of patients at Patan Hospital from Chalnakhel locality in Dakchinkali municipality, Kathmandu with AUFI from June 10 to July 1, 2016. Focused group discussion with local inhabitants and the epidemiological curve of febrile patients at local primary health care centre confirmed the outbreak. The household-survey was conducted in the area with questionnaire administered on patients to characterize their illnesses and their medical records were reviewed. A different set of questionnaire was administered on the patients and controls to investigate the association with common risk factors. Water samples were collected and analyzed microbiologically.

**Results:**

Eighty one patients from 137 households suffered from febrile illness within 6 weeks window before the investigation. All the 67 sampled patients with acute fever had a generalized illness without a discernible focus of infection. Only 38% of the patients had received a clinical diagnosis while the rest were treated empirically without a diagnosis. Three patients had blood culture confirmed enteric fever. Forty-two (63%) patients had been administered antibiotics, most commonly, ofloxacin, cefixime or azithromycin with a mean fever clearance time of 4 days. There was no definite association between several risk factors and fever. Fecal contamination was noted in tap water samples.

**Conclusion:**

Based on the pattern of illness, this outbreak was most likely a mixture of self-limiting viral infections and enteric fever. This study shows that even in the absence of a confirmed diagnosis, a detailed characterization of the illness at presentation and the recovery course can suggest the diagnosis and help in formulating appropriate recommendation for treatment and control.

## Background

Fever is a common presentation in district hospitals and primary health facilities of developing countries [1]. For many such febrile illnesses, it is difficult to pin-point an aetiology based on history and clinical examination alone because of a non-specific presentation, apart from a documented or undocumented rise of temperature, and lack of a localizing physical sign. In the absence of reliable point-of-care diagnostic tests to differentiate the aetiology in resource poor settings, ‘fever’ itself is established as a diagnosis rather than a symptom and treated empirically with antimicrobials [2]. Such acute ‘undifferentiated’ febrile illnesses (AUFI) are often attributed to self-limiting viral infections in the industrialized countries, but in the developing world, they are more commonly caused by malaria, dengue, enteric fever, rickettsial illness, leptospira and other agents [3].

AUFIs are common occurrences in Kathmandu, Nepal and most commonly caused by mildly virulent self-limiting viral infections [4]. However, outbreaks of more severe form of viral infection like pandemic influenza A have been reported [5, 6]. Enteric fever remains endemic in Kathmandu and has a potential for large outbreaks due to high population density, water contamination, and inadequate hygiene and sanitation [7]. Outbreaks of significant magnitude have been reported from elsewhere in Nepal in the past [8, 9]. Rickettsial infections, both murine typhus and scrub typhus have been reported as common causes of AUFI in Nepal [10, 11]. A large outbreak of scrub typhus from mid-western region of Nepal has been reported after the major earthquake of 2015 [12]. Due to a lower reported prevalence of rickettsial illnesses, a clinical presentation resembling enteric fever and a lack of a reliable point-of-care diagnostic test, these illnesses are also presumed and treated as enteric fever [13].

We report an epidemiologic, environmental and clinical-microbiologic investigation of a suspected outbreak of acute febrile illness in order to characterize the nature of the outbreak and compare the characteristics of cases with controls to determine any risk factors associated with the outbreak.

## Methods

### Outbreak area

Bosan is a sub-urban area situated in Dakchhinkali Municipality, previously Chalnakhel Village Development Committee, on the northern base of Champadevi Hill in KathmanduValley, Province No 3, Nepal. The area is connected to the Kathmandu city by road transport but the lack of regular public transport services makes it a difficult connection to the city. Apart from this, the village is located at the top of a hill and people have to walk down the road for about 30 min to reach the road for transport services. People are mostly dependent on agriculture for living. Rearing of cattle and poultry is common and the products are used for household consumption or sold in the market.

People use tap water for drinking, cooking and cleaning purposes. The water is supplied from a reservoir tank built on a hill, which stores water from a river source nearby. There is no provision for regular water purification. No surveillance mechanism is in place to ensure the quality of water at source, reservoir and distribution level. Primary Health Care Centre (PHCC) is located at around 45 min walking distance from the locality and is the nearest public health facility. The facility runs outpatient general clinics and provides essential drugs free of cost.

### Case definition

We defined a case as illness in a resident of the Bosan area in Dakchinkali municipality, Kathmandu, who had an episode of febrile illness of onset between May 28 and July 8, 2016. For households with more than one member having febrile illness, one member was conveniently selected as a case. Controls were selected by convenient sampling from the consecutive households with no member in the household with febrile illness of onset between May 28 and July 8, 2016. Matching of cases and controls was not done.

We categorized the illness into two categories on the basis of duration of febrile illness. We classified the cases with illness of duration of seven days or more as ‘Long acute febrile illness’. Patients with febrile illness of duration less than seven days were categorized as ‘Short acute febrile illness’. We described the response to the antimicrobial as ‘Quick antimicrobial response’ if patients under antimicrobial therapy had a subjective disappearance of symptom of fever within 3 days of the start of antimicrobial therapy. The response was defined as ‘Late antimicrobial response’ if patients under antimicrobial therapy experienced fever for longer than 3 days after the initiation of antimicrobial treatment.

We defined Fever Clearance Time (FCT) as the time from the first dose of antimicrobial therapy till the time of subjective disappearance of symptom of fever as reported by patient.

### Clinical and epidemiological investigation

An unusually large number of patients with acute febrile illness from the Bosan area were noticed in Patan Hospital between 19 and 30 June, 2016. *Salmonella* Typhi was isolated in blood culture of two patients from the area. We suspected of an outbreak of acute febrile illness and initiated an investigation. On the 4th of July, a team of investigators visited the locality and confirmed the outbreak by a focused group discussion and interviews involving community members and local health workers. We plotted the epidemiological curve of febrile patients at the local PHCC.

The household survey was done on 8th and 9th July 2016 in the area of suspected outbreak by visiting consecutive households. We administered a questionnaire to the cases and attempted to characterize their illness in terms of the demographic characteristics, symptoms they had, duration of their illness, presumptive diagnosis, treatment received and response to treatment, course of the illness and the outcome. Their medical records were reviewed. We also administered a different set of questionnaire to both the case and the control groups in an attempt to determine the common risk factors of AUFI in the outbreak setting. We asked questions related to the risk factors associated with water, sanitation and hygiene, common eating habits and exposure to domestic animals.

### Laboratory investigation

We collected blood samples from acutely ill patients at the time of the household survey and subjected them to blood culture and Widal’s agglutination test. Blood samples for culture were inoculated into commercially available BD BACTEC blood culture bottles according to manufacturer’s instruction and were immediately transported to National Public Health Laboratory in Kathmandu. *Salmonella* Typhi was identified after subculturing and stereotyping according to standard protocols. Water samples used for drinking, cooking and cleaning purposes were collected from piped water supply of randomly selected households. The samples were subjected to microbiological analysis for fecal contamination by coliform count and residual chlorine content.

### Data collection and analysis

Data was collected on a paper questionnaire. The data entry was done using Microsoft Excel 2010. Odds ratio was calculated with 95% confidence interval. The statistical significance was set at *p* < 0.05.

### Interventions

After the investigation, a team from the Epidemiology and Disease Control Division, District Public Health Office, Kathmandu and Immunization Preventable Diseases Unit was deployed on the dates of 17 to 21 July 2016. This team distributed 18,000 tablets of ‘Aquatabs’ and imparted information relating to water treatment, sanitation and hygiene. On 18 July 2016 interaction program with local inhabitants was carried out in local secondary school to raise awareness on protection of oneself and the community from acute febrile illnesses including typhoid fever. The local water consumers’ committee was also asked to make sure that the piped water be treated before distribution.

## Results

### Clinical and epidemiological investigation

**Fig. 1:**
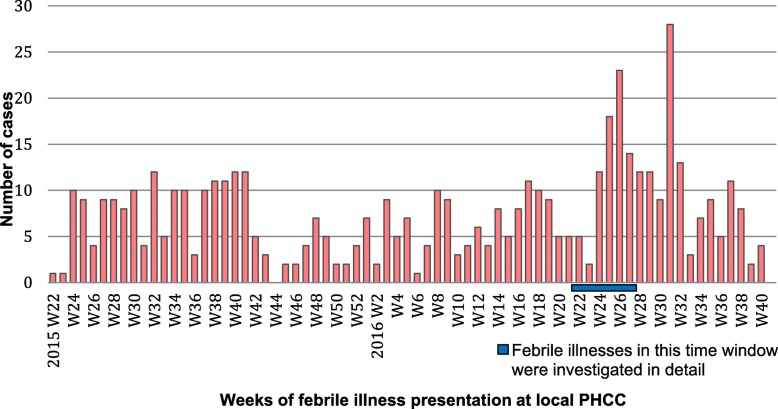
Epidemic curve showing patients with febrile illness at the primary health care centre in the outbreak location

The epidemiological curve of febrile patients at the local PHCC pointed to the outbreak in the area (Fig. 1).

Among 137 households screened for febrile illness by household survey, 81 individuals were found to have an episode of febrile illness in 67 households between May 28 and July 8, 2016.

We interviewed 67 cases each from different households. Other than generalized illness, none had a discernible focus of infection. Thirty-five cases (52.2%) were females. The median age of the cases was 17 years. The demographic and clinical characteristics of the cases are illustrated in Table 1.

**Table 1:** Demographic and clinical characteristic of cases

Demographics	n (%)
Female	35 (52.2)
Median age	17 years
Characteristic of Febrile illness	
Long acute febrile illness	18
Short acute febrile illness	13
Ongoing febrile illness	36
Mean febrile duration in patients with subsided fever	7.19 days
Symptoms	
Chills	25 (37.3)
igor	26 (38.8)
Nausea	14 (20.9)
Vomiting	18 (26.8)
Loose stool	26 (38.8)
Abdominal pain	24 (35.8)
Cough	18 (26.8)
Coryza/Running nose	15 (22.3)
Headache	33 (49.2)
Body ache	22 (32.8)
Joint ache	12 (17.9)
Weakness	25 (37.3)
Rashes	4 (5.9)
Antibiotics use and Response	
Received antibiotics	42/67 (63)
Quick antimicrobial response	6 (14.3)
Late antimicrobial response	19 (45.2)
Receiving antimicrobial at the time of survey	17 (40.5)
Mean FCT in patients with subsided fever (25/42)	4.03 days

Thirty one (31) patients had recovered from the infection at the time of the survey among which 18 (58.1%) had ‘long acute febrile illness’ and 13 (41.9%) had ‘short acute febrile illness’. Among the 36 patients with ongoing febrile illness, 19 (52.8%) cases were still within the ‘short acute febrile illness’ window and 17 (47.2%) were ‘long acute febrile illness’.

Among 42 patients who had received antibiotics, 25 (59.5%) patients had recovered from the illness at the time of survey with ‘quick antimicrobial response’ in 6 patients and ‘late antimicrobial response’ in 19 patients. Seventeen (40.5%) patients were still receiving antimicrobials at the time of the survey. Mean fever clearance time in patients with subsided fever (25/42 patients receiving antibiotic) was 4.03 days.

Thirty-four patients (51%) received care for their illness from private clinics and local pharmacies, 25 (37%) patients from hospitals and PHCC; and 8 patients (12%) didn’t seek any medical care. Among 42 patients who had received antimicrobials for their illness, only 16 patients (38%) received a clinical diagnosis, with 13 patients (30%) diagnosed as enteric fever. Twenty-six patients (61.9%) received an antimicrobial without any diagnosis. The diagnosis received by patients based on their clinical and laboratory parameters in patients receiving antimicrobial is illustrated in Table 2.

**Table 2:** Diagnosis made on clinical and laboratory parameters of patients with acute febrile illness

Diagnosis in patients receiving antimicrobials	*N* = 42 (%)
Enteric Fever	13 (30.9)
Upper Respiratory Tract Infection	1 (2.4)
Acute Gastroenteritis	1 (2.4)
Scrub Typhus	1 (2.4)
No diagnosis	26 (61.9)

Forty-two patients out of 67(62.6%) had been treated with various antibiotics, most commonly, ofloxacin, cefixime or azithromycin. Other antimicrobials used in the treatment were amoxicillin, ciprofloxacin, cotrimoxazole, doxycycline, cefpodoxime and metronidazole. Table 3 illustrates the various antimicrobials used in 13 patients who received the diagnosis of enteric fever and 26 patients who received antimicrobial without any clinical diagnosis.

**Table 3:** Table showing various antibiotics received by patients with a clinical diagnosis of enteric fever and patients without any provisional diagnosis

Antimicrobial used	Clinically diagnosed as enteric Fever (*N* = 13)	No clinical diagnosis (*N* = 26)
Azithromycin	3	3
Azithromycin + cefixime	1	1
Cefixime	2	4
Amoxycillin + Cefixime	1	
Ofloxacin	3	5
Ofloxacin + Metronidazole	2	3
Amoxycillin		4
Azithromycin + Metronidazole		1
Ciprofloxacin + Metronidazole		2
Cotrimoxazole		1
Cefpodoxime		1
Doxycycline		1
Unknown antibiotic	1	

There was no statistically significant association between various risk factors and the development of febrile illness. The odds of various risk factors in cases and controls are illustrated in Table 4. The odds of febrile illness was higher in the cases with animal exposure in their households and history of eating outside their usual eating place in home, office/school cafeteria etc. but due to the small sample sizes under consideration no statistical significance was detected.

**Table 4:** Table showing proportion of cases and controls exposed to various risk factors

Outcome measures	Cases	Controls	Odds ratio(95% CI)	*P*-value
No hand washing	1/67	1/70	0.95 (0.05–15.60)	0.975
No treatment of drinking water at household level	50/67	48/70	0.74 (0.35–1.56)	0.432
Unusual eating places	7/67	3/70	2.60 (0.64–10.53)	0.166
Animal contacts	34/67	25/70	1.85 (0.93–3.67)	0.076

### Laboratory investigations

Among 14 blood cultures performed in patients with ongoing febrile illness at the time of the investigation, one tested positive for *Salmonella* Typhi which was sensitive to chloramphenicol, cotrimoxazole, ciprofloxacin and ceftriaxone, resistant to nalidixic acid and intermediately sensitive to ampicillin. The remaining showed no growth after 72 h of incubation at 37 C. Seven samples from these patients tested positive for Widal’s test.

All the water samples tested from the piped source of five different households showed a presumptive count of faecal *E coli* greater than 180 per 100 ml. Residual chlorine in these samples ranged from 0.02 to 0.07 mg/L in four of the five samples and 0.23 mg/L in one sample with a reference range specified as 0.1–0.2 mg/L.

### Evaluation of interventions

A qualitative interview of the stakeholders revealed a decline in the numbers of patients with febrile illness in the weeks following the interventions. The epidemic curve based on the nearest Primary Health Care centre revealed yet another week with a larger spike in febrile illness which dropped down in consecutive weeks (Fig. 1). No systematic follow-up evaluation of interventions was not done.

## Discussion

This study was designed with two objectives. Firstly, we attempted to discern any risk factors attributable to the febrile illnesses present in outbreak area, and secondly we aimed to clinically characterize the illness based on the symptoms, laboratory features and response to administered treatments.

The selection of water, sanitation and food habits as risk factors worthy of examination was prompted by the fact that this outbreak was initially noticed when two patients with blood culture proven enteric fever (*Salmonella* Typhi) presented to Patan Hospital from the study area. The choice of examining animal exposure was made because many such outbreaks of acute febrile illness are due to zoonotic viral infections. The occurrence of febrile illness with no association with these risk factors could be attributed to differential contamination or the immune status of the subjects.

We attempted to classify AUFIs into long acute febrile illness and short acute febrile illness. The illness with duration of ≥7 days is likely to be of bacterial origin and the illness of < 7 days which resolved without antimicrobial treatment could be attributed to self-limiting viral infections. The delayed response to antimicrobial therapy points out to more serious infection, inappropriate antimicrobial therapy and/or potential antimicrobial resistance to the administered drug. The early antimicrobial response is generally obtained in patients when the bacterial illness is susceptible to the administered antimicrobial or in patients with self-limiting viral illness where antimicrobial was empirically administered in the line of enteric fever which is one of the major causes of AUFI in this setting.

The assumption of an outbreak of a mixture of viral and bacterial infection was supported by the findings of greatly variable duration of fever and associated symptoms, variation in fever clearance times after antimicrobial treatment, non-uniformity in the response to treatment and spontaneous recovery without treatment in a subset of patients. Self-limiting air borne viral influenza A infections, and food and water borne bacterial infections including *Salmonella* Typhi and *Salmonella* Paratyphi A are endemic in the study setting and exhibit similar seasonality, with the peak incidence coinciding during the monsoon seasons [7, 14]. The present study possibly is a representative of such phenomena of mixed outbreaks of viral and bacterial infections. These infections have similar initial presentation, a unique clinical course and treatment response. Therefore, making a proper diagnosis is crucial for patient management, outbreak control and antimicrobial stewardship [15].

Tackling the syndrome of fever requires a sound understanding of etiologies prevalent in a given epidemiological setting, accessibility of reliable point-of-care diagnostic tests and the treatment protocol with appropriate drugs [16, 17]. In the absence of robust epidemiological data and reliable point-of-care diagnostics, a large number of patients in the outbreaks of AUFI in enteric fever endemic settings such as Kathmandu, Nepal are empirically diagnosed and treated as enteric fever [18, 19]. It is a common practice to initiate antimicrobials with minimal effort to establish a specific diagnosis [15]. This can lead to prolonged fever clearance time, treatment failures, and antimicrobial resistance. Laboratory tests to differentiate these infections are not available outside hospital setting and available rapid tests for non-malarial causes of fever are largely insufficient [20, 21]. The detailed characterization of the illness at presentation and clinical course can suggest appropriate diagnosis and help formulate recommendations for treatment and control efforts [16, 22].

How such illness events should be approached from the perspective of disease control is a vexing question. WHO has formulated the guidelines for the management of common illnesses in resource poor settings for children, and adolescent and adults [23, 24]. The development of protocols for the investigations and management of AUFIs in hospital settings of South Asia have been attempted [25]. However these protocols are insufficient to approach the diagnosis and management of febrile illness within outbreaks in developing countries where large number of cases is treated outside healthcare facilities without clinical expertise and reliable diagnostic tests at the point-of-care [21]. An epidemiological approach to an outbreak and protocol for the diagnosis and treatment is critical for the management of patients and stopping disease transmission. Owing to large heterogeneity in the etiologies over time and space, geographically explicit protocols taking into account of seasonality would improve the treatment outcomes and accelerate control efforts [21, 26]. A routine real-time reporting of surveillance data with advanced computational methods for the epidemiological analysis will help to rapidly identify an outbreak and the factors associated with it.

The healthcare delivery is largely dependent on informal providers in developing countries [27]. These providers although highly engaged with the communities they serve often are inadequately trained to address the healthcare problems. Appropriate training of these informal providers where the clinical expertise are out-of-reach, on specific health conditions can increase correct case management rates and improve the treatment outcomes [28].

Given the limited time and resources to investigate the outbreak for its early containment and control, there were the following limitations. Controls were conveniently selected and matching for the possible confounders while selecting the controls was not performed which might have reduced the robustness of comparison of risk factors between cases and controls. The investigation suggested the outbreak to be a mixture of bacterial and viral infections, however couldn’t make a aetiological differentiation of the possible viral pathogens. Viral PCRs or cultures are hard to obtain in a setting such as the one for the study, besides for many common and mild infections it doesn’t change the treatment outcome. The study emphasizes the diagnostic challenge with both viral and bacterial infections as one of the key points in approaching outbreaks such as this one in a setting with limited resources. In addition, no systematic follow-up evaluation of community interventions was performed. However, qualitative interviews with stakeholders revealed a decline in number of patients in the weeks following interventions.

We have reported this outbreak because outbreaks of febrile illness are common occurrences in district hospitals and primary health centers in Nepal and south Asia. This investigation report gives insight into both the epidemiological management of the outbreak and clinical treatment of individual patients. In the absence of reliable diagnostic tools at the point-of-care, it is important to properly characterize such outbreaks in terms of etiology, environmental risk factors, clinical presentation, laboratory findings and outcome. We recommend that a specific plan of action be put in place with the probable questionnaire, sampling method and sample collection and testing protocol so that a rapid investigation may be carried out to a plan should such an event occur again. Community health workers, hospitals and physicians, public health officials and laboratory workers must remain prepared with the necessary investigational tools for future large unexpected outbreaks of febrile illness.

## Conclusion

The findings of the investigation suggest that the outbreak was potentially a mixture of viral infections and enteric fever, and empirical use of antimicrobial drugs for all causes of fever is a challenge. No etiological differentiation of cause was possible, except for three blood-culture confirmed enteric fever patients, due to the absence of reliable diagnostic tests at the point-of-care. No associated specific risk factor for the febrile illness patients related to water, sanitation, eating habits and animal exposure was found. This study highlights the importance of detailed characterization of illnesses in terms of etiology, environmental risk factors, clinical presentation, laboratory findings and outcome in suggesting appropriate diagnosis and treatment of individual patients and epidemiological management of outbreak. Outbreak preparedness and response plan with necessary investigation tools are urgently required for early containment and control should such outbreaks occur in future.

## Data Availability

All dataset generated and analyzed during the study are not publicly available but are available from the corresponding author upon a reasonable request.

## Abbreviations

AUFI: Acute Undifferentiated Febrile Illness
FCT: Fever Clearance Time
PHCC: Primary Health Care Centre
PUO: Pyrexia of Unknown Origin
WHO: World Health Organization
